# Age-Dependent Variations in the Distribution of *Aeromonas* Species in Human Enteric Infections

**DOI:** 10.3390/pathogens14020120

**Published:** 2025-01-28

**Authors:** Adhiraj Singh, Fang Liu, Christopher Yuwono, Michael C. Wehrhahn, Eve Slavich, Alexandra M. Young, Sarah K. T. Chong, Alfred Chin Yen Tay, Stephen M. Riordan, Li Zhang

**Affiliations:** 1 School of Clinical Medicine, University of New South Wales, Sydney, NSW 2052, Australia; adhiraj.singh@student.unsw.edu.au; 2 School of Biotechnology and Biomolecular Sciences, University of New South Wales, Sydney, NSW 2052, Australia; fang.liu@unsw.edu.au (F.L.); c.yuwono@student.unsw.edu.au (C.Y.); alexandra.young@unsw.edu.au (A.M.Y.); sarah.chong@student.unsw.edu.au (S.K.T.C.); 3 Douglass Hanly Moir Pathology, a Sonic Healthcare Practice, 14 Giffnock Ave, Macquarie Park, NSW 2113, Australia; mwehrhahn@dhm.com.au; 4 Stats Central, Mark Wainwright Analytical Centre, University of New South Wales, Sydney, NSW 2052, Australia; eve.slavich@unsw.edu.au; 5 Helicobacter Research Laboratory, Marshall Centre for Infectious Diseases Research and Training, School of Biomedical Sciences, University of Western Australia, Perth, WA 6009, Australia; alfred.tay@uwa.edu.au; 6 Gastrointestinal and Liver Unit, Prince of Wales Hospital, University of New South Wales, Sydney, NSW 2033, Australia; stephen.riordan@health.nsw.gov.au

**Keywords:** *Aeromonas*, gastroenteritis, *Aeromonas veronii*, *Aeromonas caviae*, *Aeromonas hydrophila*, *Aeromonas dhakensis*

## Abstract

*Aeromonas* species are enteropathogens that cause gastroenteritis with a unique three-peak infection pattern related to patient age. The contributions of individual *Aeromonas* species to age-related infections remain unknown. Multi-locus sequence typing (MLST) was performed to determine the species of *Aeromonas* strains from Australian patients with gastroenteritis. Public database searches were conducted to collect strains of enteric *Aeromonas* species, identified by either MLST or whole genome sequencing with known patient age. Violin plot analysis was performed to assess *Aeromonas* infection distribution across patients of different ages. Generalized additive model (GAM) analysis was employed to investigate the relationship between *Aeromonas* species and patient age. A total of 266 strains of seven *Aeromonas* species met the selection criteria, which were used for analyses. The violin plots revealed distinct patterns among individual *Aeromonas* species in relation to patient age. The GAM analyses identified a significant association between *Aeromonas* species and patient age (*p* = 0.009). *Aeromonas veronii* (153 strains) showed the highest probability of infection in most ages, particularly among young adults. *Aeromonas caviae* (59 strains) is more common in young children and adults over 60 years of age. The probability of infection for *Aeromonas hydrophila* (34 strains) and *Aeromonas dhakensis* (9 strains) was generally low, there was a slight increase in individuals aged 50–60 for *A. hydrophila* and over 60 years for *A. dhakensis*. These findings provide novel evidence of the varied contributions of different *Aeromonas* species to human enteric infections related to patient age, offering valuable insights for epidemiology and clinical management.

## 1. Introduction

*Aeromonas* species are facultative anaerobic, Gram-negative, rod-shaped bacteria that are commonly found in aquatic environments, including freshwater and seawater. These bacteria are significant pathogens in aquatic animals, such as fish [1]. Some *Aeromonas* species are also important human pathogens, contributing to an increasing burden of infectious diseases globally. *Aeromonas* species have been reported to cause a broad range of human infections in both immunocompetent and immunocompromised individuals, including infections in the gastrointestinal tract, wounds, soft tissues, bloodstream, and various other extra-intestinal illnesses.

While over 10 *Aeromonas* species have been recognized as human pathogens, more than 90% of human *Aeromonas* infections are caused by 4 primary species: *Aeromonas caviae*, *Aeromonas veronii*, *Aeromonas dhakensis*, and *Aeromonas hydrophila* [2]. There is growing evidence suggesting that different *Aeromonas* species may exhibit variability in clinical manifestations, with some species causing relatively milder, self-limiting disease, while others result in severe systemic or extra-intestinal infections [2,3,4,5,6,7]. For example, previous research has highlighted that monomicrobial *A. dhakensis* bacteremia is an independent risk factor for mortality [8,9]. This diversity is also reflected in the expression of virulence factors and antimicrobial resistance profiles, which vary significantly between species and even between strains within the same species, suggesting different pathogenic potentials and clinical outcomes [8,9,10,11,12,13,14].

Biochemical tests and matrix-assisted laser desorption ionization–time-of-flight (MALDI-TOF) mass spectrometry are commonly used by diagnostic laboratories to identify *Aeromonas*. While these methods can reliably identify *Aeromonas* at the genus level, they are unable to accurately identify *Aeromonas* to species level [15,16,17,18]. In recent years, molecular techniques such as multi-locus sequence typing (MLST), which targets multiple housekeeping genes, and whole genome sequencing have been used by research groups for precise *Aeromonas* species identification.

Among the different types of human infections caused by *Aeromonas* species, gastrointestinal infections are the most common [1,19]. A recent study has shown that *Aeromonas* species were the second most frequently isolated bacterial enteropathogens in Australian patients with gastroenteritis [20]. Epidemiological data also showed that *Aeromonas* gastrointestinal infections exhibit distinct age-related patterns, with infection peaks occurring in young children, young adults, and individuals over 50 years old [20]. These age-related trends highlight the potential differences in host susceptibility and clinical presentation of infection in different age groups. For example, *Aeromonas* species are the most common bacterial gastroenteric pathogen in children younger than 18 months [20]. However, while age-related infection peaks have been well documented, the contribution of individual *Aeromonas* species to these age-related infections remains unclear.

The aim of this study is to investigate the association between different *Aeromonas* species and patient age in human gastrointestinal infections. Understanding whether specific *Aeromonas* species are linked to age-related patterns of gastrointestinal infections is both epidemiologically and clinically relevant.

## 2. Materials and Methods

### 2.1. Preparation of Aeromonas Strains for Analysis

Australian strains: 39 *Aeromonas* isolates, isolated from the fecal samples of patients with gastroenteritis, were defined to species level in this study. These *Aeromonas* isolates were provided by Douglass Hanly Moir (DHM) pathology. An additional 53 Australian *Aeromonas* enteric isolates, isolated from patients with gastroenteritis, which were defined to species level in our previous studies were also included in the analysis [12,21,22,23].

DNA was extracted from each of the 39 *Aeromonas* isolates using the Qiagen© DNA Extraction and Purification Kit and subjected to whole genome sequencing using the Illumina MiSeq Personal Sequencer at the Helicobacter Research Laboratory, University of Western Australia. For *Aeromonas* species identification using the MLST method, the nucleotide sequence of seven housekeeping genes, namely, *gyrB*, *rpoD*, *gyrA*, *recA*, *dnaJ*, *dnaX*, and *atpD*, were extracted from the genome sequences and used for comparison with those from known *Aeromonas* species in the National Center for Biotechnology Information (NCBI) database for *Aeromonas* species identification, as previously described [21]. The sequences of housekeeping genes of these strains have been submitted to Genbank under the following accession numbers: PQ537383-PQ537413, PQ537414-PQ537444, PQ537445-PQ537475, PQ537476-PQ537506, PQ537507-PQ537537, PQ537538-PQ537568, PQ537569-PQ537599, PQ537600-PQ537606, PQ537607-PQ537613, PQ537614-PQ537620, PQ537621-PQ537627, PQ537628-PQ537634, PQ537635-PQ537641, PQ537642-PQ537648, and PQ622943-PQ622949.

**Figure 1:**
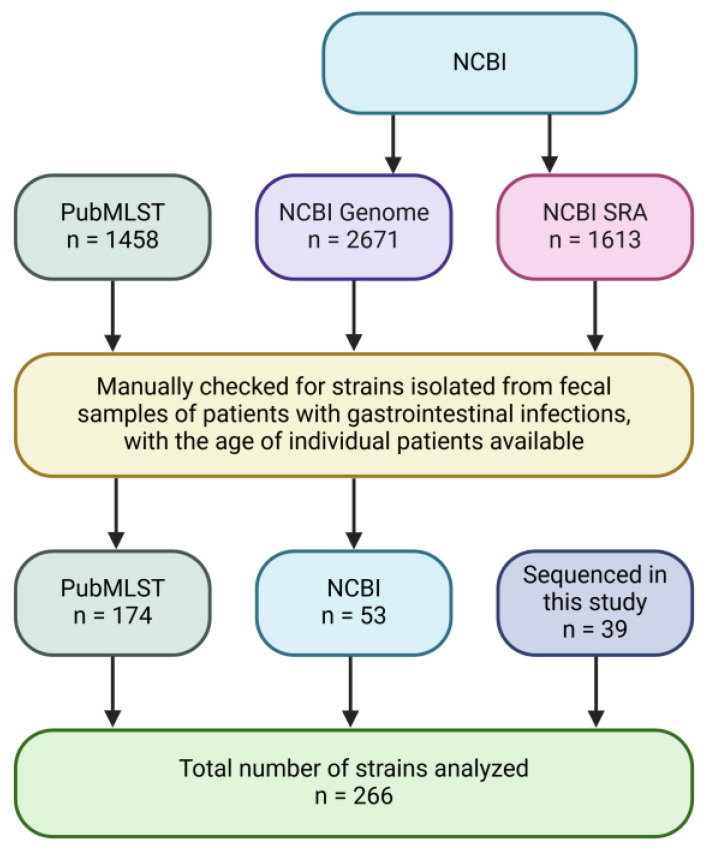
Flowchart of *Aeromonas* strain collection. A total of 266 strains were included for analysis; these were retrieved from the PubMLST database, NCBI genome database, and NCBI SRA database, or were sequenced in this study.

Strains in public databases: Searches for the enteric strains of *Aeromonas* species isolated from patients with gastroenteritis were performed, as described in Figure 1. *Aeromonas* genomes in the NCBI genome database and the Sequence Read Archive (SRA) were manually checked for strains that satisfied the selection criteria. All *Aeromonas* strains in the PubMLST database were also checked.

The selection criteria required that *Aeromonas* strains be isolated from the fecal samples of patients with gastroenteritis, that the sample’s country of origin and the patient’s age be specified, and that the strain be identified to a species level using either whole genome sequencing or MLST typing. These molecular methods accurately define *Aeromonas* to the species level [1].

### 2.2. Visualization of the Distribution of Aeromonas Species Among Patients of Different Ages

The age distribution of strains of *Aeromonas* species that satisfied the selection criteria were visualized with violin plots, generated using the ggplot2 package in R (version R4.4.1). In this analysis, *Aeromonas* species with five strains or fewer were grouped together as “Rare Species”, while those with more than five strains were analyzed as individual groups.

### 2.3. Generalized Additive Model Analysis

Multinomial generalized additive model (GAM) analysis was conducted using R, with the mgcv package (version 1.9-1) [24,25].

The model aims to predict the proportion of infections by each *Aeromonas* species among patients of varying ages, within different countries, who are infected with *Aeromonas*. The response variable was the *Aeromonas* species of gastrointestinal infection, with the predictor variables of patient age (a continuous effect) and the country of origin (a categorical effect). A logit-link function was used, and thin plate splines were used to smooth the effect of age.

To assess whether age affected the *Aeromonas* species implicated in infection, a likelihood ratio test was conducted, comparing model likelihoods with and without age data. A *p*-value of <0.05 was considered significant.

### 2.4. Examination of the Presence of Aerolysin (aerA) and Thermostable Cytotonic Enterotoxin (ast) Genes

In our previous study, we found that aerolysin and Ast toxins were absent in *A. caviae* strains isolated from patients with gastroenteritis [12]. In the study, we further examined the presence of the *aerA* and *ast* genes in 92 Australian isolates for which the genomes were available. The presence of *aerA* and *ast* genes was examined using BLASTn [26]. Query sequences of the *aerA* and *ast* genes were obtained from *A. veronii* and *A. hydrophila*, respectively.

## 3. Results

### 3.1. Aeromonas Strains Used in This Study

A total of 266 *Aeromonas* strains met the selection criteria for inclusion in this study, including 92 Australian *Aeromonas* strains, of which 39 were identified to the species level in this study, and 174 strains from China were obtained from public databases (Table 1 and Figure 1). Four strains from the USA and two strains from Tanzania also met the selection criteria; however, the sample sizes from these two countries were too small and, therefore, were not included in the analysis. Additional details on the strains used in this study are provided in Supplementary Table S1.

### 3.2. Distribution of Aeromonas Species Among Patients of Different Ages with Gastroenteritis

**Figure 2:**
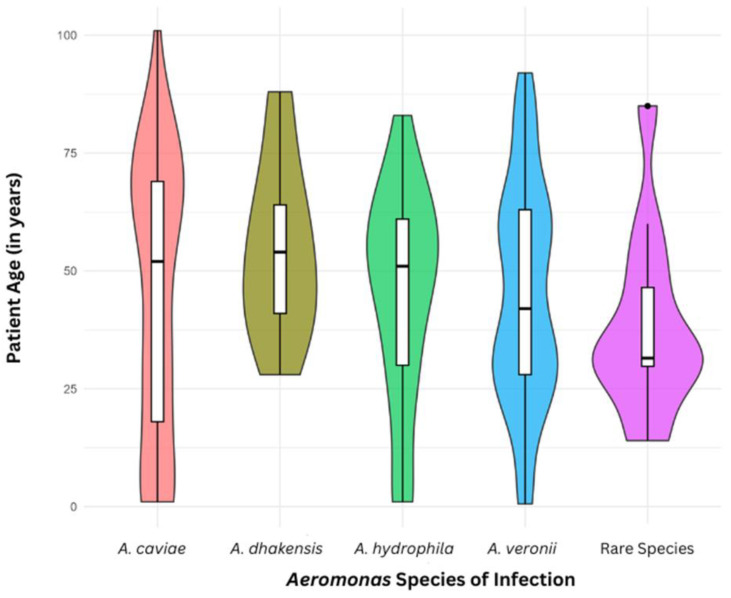
Violin plot analysis of *Aeromonas* species in patients of different ages. The width of the plots at any given point depicts the frequency of infections at that specific age. A total of 266 *Aeromonas* strains from patients with gastrointestinal infections were included for analysis, including *A. caviae* (59 strains), *A. dhakensis* (9 strains), *A. hydrophila* (33 strains), and *A. veronii* (153 strains), along with a Rare Species group containing 5 *A. trota* strains, 4 *A. rivipollensis* strains, 2 *A. media* strains, and 1 *A. allosaccharophila* strain. The 266 strains were from Australia (92 strains) and China (174 strains).

A violin plot was created to visualize the distribution of patient age with respect to the different *Aeromonas* species isolated from patients with gastrointestinal infections (Figure 2). The frequency of infections varied among the *Aeromonas* species with respect to patient age. Different *Aeromonas* species exhibited distinct age distribution patterns, none of which followed a linear distribution. Given this finding, a GAM model was chosen for further analysis.

### 3.3. GAM Analyses

**Figure 3:**
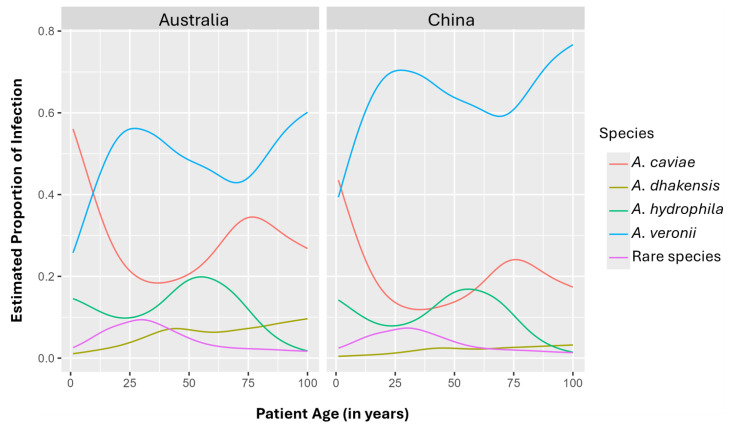
Generalized additive modeling analysis of *Aeromonas* species in response to patient age in gastrointestinal infections within specific countries. The analysis revealed a significant association between patient age and infection due to *Aeromonas* species (*p* = 0.009). A total of 266 *Aeromonas* strains from patients with gastrointestinal infections were included for analysis, including *A. caviae* (59 strains), *A. dhakensis* (9 strains), *A. hydrophila* (33 strains), and *A. veronii* (153 strains), along with a Rare Species group containing 5 *A. trota* strains, 4 *A. rivipollensis* strains, 2 *A. media* strains, and 1 *A. allosaccharophila* strain. The 266 strains were from Australia (92 strains) and China (174 strains).

The likelihood ratio tests showed a significant association between patient age and the *Aeromonas* species of infection (*p* = 0.009) (Figure 3).

*A. veronii* was the most prevalent species across most patient age groups, with the exception of young children (Figure 3). In young adults around 25 years old, the estimated proportion of infections that were *A. veronii* was 70% in Chinese patients and approximately 55% in Australian patients, while the proportion of infections caused by all other species was 20% or below. Although *A. veronii* remained the dominant infective species, the proportion of *A. veronii* infections declined in individuals aged 50 years old and above, likely due to an increase in infections caused by other *Aeromonas* species (Figure 3).

Among young children, the most common infection was *A. caviae*, accounting for close to 60% of infections among Australian patients and 40% of infections in Chinese patients (Figure 3). As a proportion of all infections, *A. caviae* decreased as the children grew older, reaching its lowest point in young adults, where the estimated proportion was below 20%. However, the proportion of infections due to *A. caviae* infection began to increase again in individuals who were 50 years old or above, although it did not reach the level observed in young children (Figure 3).

The overall proportion of infections due to *A. hydrophila* and *A. dhakensis* was low. However, there was a slight increase in the probability of *A. hydrophila* infection in individuals who were 50–60 years old and a slight increase in the probability of *A. dhakensis* in individuals who were 60 years old and above (Figure 3).

### 3.4. The Presence of aerA and ast Genes in Different Aeromonas Species

The presence of *aerA* and *ast* genes was examined in 92 strains, collected from patients with gastroenteritis in Australia, for which the genomes are available (Table 2 and Supplementary Table S1). These two genes were both absent in the *A. caviae* strains. The *aerA* gene was present in all *A. dhakensis* strains (6/6), half of the *A. hydrophila* strains (6/12), and all the *A. veronii* strains examined (45/45), while the *ast* gene was absent in the *A. dhakensis* and *A. veronii* strains but present in almost all *A. hydrophila* strains examined (11/12).

## 4. Discussion

*Aeromonas* species are common and significant contributors to gastroenteritis in the Australian population, with infections being more prevalent in young children, young adults, and individuals over 50 years old [13]. *Aeromonas* enteric infections have also been reported worldwide, with multiple *Aeromonas* species being implicated in human gastrointestinal illness. In this study, we have analyzed the impact of various *Aeromonas* species on enteric infections across different age groups of patients.

Biochemical tests, either alone or coupled with MALDI-TOF technology, have been used to identify *Aeromonas* in diagnostic laboratories. However, these methods are unable to accurately identify *Aeromonas* to the species level [15,16,17]. Molecular methods, such as whole genome sequencing and MLST, based on multiple housekeeping genes can accurately identify *Aeromonas* species [17]. Some earlier studies reported *A. hydrophila* as the most frequently isolated species found in patients with gastroenteritis [27,28]. However, more recent studies employing molecular methods to identify *Aeromonas* species have shown that *A. veronii* and *A. caviae* are responsible for the majority of *Aeromonas* gastrointestinal infections [20,21]. Currently, molecular species typing is not a routine practice in diagnostic laboratories for the identification of *Aeromonas* species, due to its labor-intensive nature. Future studies are needed to improve the MALDI-TOF technology or to develop streamed molecular methods that enable diagnostic laboratories to accurately diagnose *Aeromonas* isolates at the species level.

Accurate identification of *Aeromonas* species is crucial for our study. All Australian *Aeromonas* strains used in this study were determined to the species level by MLST using seven housekeeping genes, which were extracted from sequenced whole bacterial genomes. For the strains obtained from the public databases, only those species defined by MLST or whole genome sequencing for which the patient’s age was known were included. Strains that met these criteria for being included in our study were from Australia and China (Table 1 and Supplementary Table S1).

A violin plot analysis revealed differences in patient age distribution across infections caused by different *Aeromonas* species and the relationship between age and the infecting species is non-linear (Figure 2). This initial observation prompted further analysis using a multinomial GAM, a statistical method suitable for analyzing non-linear trends, to determine whether specific *Aeromonas* species are associated with age in patients with *Aeromonas*-associated gastroenteritis. GAM analysis revealed a significant association between *Aeromonas* species and patient age (Figure 3).

In infected young children, *A. caviae* infections were most common, although this was not the case in young adults (Figure 3). Consistent with our findings, a recent study from Pakistan using whole genome sequencing to identify species found *A. caviae* to be the most prevalent species among *Aeromonas* pediatric gastrointestinal infections, accounting for 64% of 447 infections among children aged 0–5 years [29]. Their results, along with our own, suggest that *A. caviae* acts as an opportunistic pathogen, primarily infecting people with a weakened immune system such as young children. We also found an increased probability of *A. caviae* infection in individuals over 50 years old, further supporting this view. Unlike other *Aeromonas* species associated with human diarrheal disease, *A. caviae* lacks several key virulence factors, including aerolysin and the thermostable cytotonic enterotoxin Ast [12]. In this study, we further examined the presence of these two genes in different *Aeromonas* species isolated from patients with gastroenteritis and confirmed that these two genes are absent in *A. caviae* strains (Table 2 and Supplementary Table S1). As a result, the individual’s immune system likely plays a crucial role in determining the clinical outcomes of *A. caviae* gastrointestinal infection, whether symptomatic or asymptomatic. Some previous studies have reported a higher prevalence of *Aeromonas* species in the fecal samples of healthy individuals compared with those with diarrhea [30]. However, these studies did not specify which *Aeromonas* species were present in these healthy individuals. Our findings suggest that future epidemiological studies on *Aeromonas* gastrointestinal infections should accurately identify *Aeromonas* isolates to the species level using appropriate molecular methods and correlate the specific species with patient age. We previously reported a peak in *Aeromonas* enteric infection in children under 2 years old in the Australian population [20]. Our current study suggests that *A. caviae* is a major contributor to this peak. Our previous study also revealed that there was a sharp rise in *Aeromonas* gastrointestinal infection in Australian children aged 6–12 months, as detected by real-time quantitative PCR [20]. This increased incidence of *Aeromonas* gastrointestinal infection continued to be observed until the children reached their second year [20]. Isolation of the specific infection sources contributing to the high *Aeromonas* gastrointestinal infection rate in this age group is essential. Such information would be invaluable for developing targeted strategies to reduce the incidence of these infections and improve public health outcomes for young children.

*A. veronii* revealed a predominant probability of infection in patients of most age groups except for young children (Figure 3). Our findings showed that *A. veronii* was the dominant species contributing to *Aeromonas* gastrointestinal infections in most age groups, particularly young adults, suggesting that *A. veronii* is a true enteric pathogen. *A. veronii* possesses multiple virulence genes, including those that are absent in *A. caviae*, and have been reported to cause severe gastrointestinal infections more frequently, further supporting its role as a true human enteric pathogen [12,22]. We previously identified a small *Aeromonas* enteric infection peak in young adults of 20–29 years old in the Australian population [13]. Our current study suggests that *A. veronii* is the primary contributor to this infection peak.

The likelihood of gastrointestinal infections caused by *A. hydrophila* and *A. dhakensis* was lower compared to *A. veronii* and *A. caviae* (Figure 3). A slight increase in *A. hydrophila* related to gastrointestinal infections was observed in patients aged 50–60 years in both the Australian and Chinese cohorts, which was potentially due to increased exposure. *A. dhakensis* infection showed an increase in individuals over 60 years old, although rates in the Chinese data were low overall. We previously reported a continuous increase in *Aeromonas* gastrointestinal infections among individuals over 50 years old; our current findings further highlight that in addition to *A. veronii*, other species such as *A. caviae*, *A. hydrophila*, and *A. dhakensis* also contributed to the growing incidence of infections in older populations.

In addition to gastrointestinal infections, *Aeromonas* species may cause other human infections such as wound infections and bloodstream infections [31,32,33]. *Aeromonas*-induced bloodstream infections more often occur in individuals over 60 years old, with approximately 39.1–66.8% of cases being associated with gastrointestinal infections [31,32]. In addition to older age, other risk factors associated with *Aeromonas* bloodstream infections have also been identified, such as malignancy and liver cirrhosis [31,32]. The 30-day mortality of *Aeromonas*-induced bloodstream infections has been reported to range from 19% to 28.8% [32,34]. Given that *Aeromonas* gastrointestinal infections are more likely to lead to bloodstream infections in older individuals, and that different *Aeromonas* species vary in their pathogenicity, it is crucial for diagnostic laboratories to identify enteric *Aeromonas* isolates to the species level, particularly in older individuals. This will ensure the application of appropriate treatment to prevent the progression of *Aeromonas*-related gastrointestinal infections into systemic infections.

In clinical practice, antibiotic therapy is not routinely applied, and the need for antibiotics when treating patients with *Aeromonas*-induced gastrointestinal infections is determined by clinicians, based on the severity of infection and the patient’s clinical condition. Treatment using ciprofloxacin and trimethoprim-sulfamethoxazole has previously been reported [35,36]. However, since *Aeromonas* isolates from fecal samples are not routinely tested for antibiotic susceptibility in many diagnostic laboratories, it is not clear whether the different *Aeromonas* species isolated from human gastrointestinal infections differ in their antibiotic resistance. Furthermore, controlled clinical trials are lacking. Consequently, the optimal antibiotic treatment regimen for such infections has not been defined.

In summary, this study is the first to report a significant relationship between the *Aeromonas* species implicated in gastrointestinal infections and patient age. Among infected young children, *A. caviae* was most common, while *A. veronii* species caused most of the infections in adults. Among patients over 50 years old, *A. veronii* remained the dominant causative species, while infections from other species such as *A. caviae*, *A. hydrophila,* and *A. dhakensis* increased as a proportion of total infections. The novel findings from our study offer valuable insights for epidemiology and clinical management. Whether *Aeromonas* enteric infections have a similar pattern in other countries warrants further investigation.

## Figures and Tables

**Table 1:** *Aeromonas* strains used for analysis in this study.

Country	Number of Strains (*n*)		
Australia	*A. caviae*	25	92
	*A. dhakensis*	6	
	*A. hydrophila*	12	
	*A. veronii*	45	
	*A. allosaccharophila*	1	
	*A. media*	1	
	*A. rivipollensis*	2	
China	*A. caviae*	34	174
	*A. dhakensis*	3	
	*A. hydrophila*	21	
	*A. veronii*	108	
	*A. media*	1	
	*A. rivipollensis*	2	
	*A. trota*	5	

**Table 2:** The presence of *aerA* and *ast* genes in different *Aeromonas* species.

Species	*aerA*	*ast*
*A. caviae* (*n* = 25)	0	0
*A. dhakensis* (*n* = 6)	6	0
*A. hydrophila* (*n* = 12)	6	11
*A. veronii* (*n* = 45)	45	0
*A. allosaccharophila* (*n* = 1)	0	0
*A. media* (*n* = 1)	0	0
*A. rivipollensis* (*n* = 2)	1	0

The presence of *aerA* and *ast* genes in 92 *Aeromonas* strains isolated from patients with gastroenteritis in Australia was examined. The query sequences of the *aerA* and *ast* genes used for BLASTn were obtained from *A. veronii* and *A. hydrophila*, respectively. *n* = the total number of strains for each *Aeromonas* species. *aerA* and *ast* genes encode for aerolysin and thermostable cytotonic enterotoxin, respectively.

## Data Availability

The accession numbers of the housekeeping genes and virulence genes are stated in the article and Supplementary Table S1.

## Supplementary Materials

The following supporting information can be downloaded at: https://www.mdpi.com/article/10.3390/pathogens14020120/s1, Table S1: List of *Aeromonas* isolates used in our analyses.
