# Supplementary material for: Age-Dependent Variations in the Distribution of Aeromonas Species in Human Enteric Infections
Source: Pathogens. 2025 Jan 28;14(2):120. doi: 10.3390/pathogens14020120 (PMC11858002; doi:10.3390/pathogens14020120)
Supplement: Supplementary file 1 [file pathogens-14-00120-s001.zip › pathogens-3364400-supplementary.pdf]

Supplementary Table S1. List of *Aeromonas* isolates used in our analyses

Serial Number	Species	Age	Country	Isolate ID	Year	*aerA*	*ast*	Source
**1**	*A. caviae*	2	Australia	Aero12	2021	N	N	[1]
**2**	*A. caviae*	4	Australia	Aero18	2022	N	N	[1]
**3**	*A. caviae*	21	Australia	A21-18	2021	N	N	[1]
**4**	*A. caviae*	24	Australia	Aero5	2021	N	N	[1]
**5**	*A. caviae*	30	Australia	AF23-12-11	2023	N	N	This study
**6**	*A. caviae*	33	Australia	Aero11	2021	N	N	[1]
**7**	*A. caviae*	41	Australia	AF23-12-10	2023	N	N	This study
**8**	*A. caviae*	52	Australia	AF23-6-2	2023	N	N	This study
**9**	*A. caviae*	58	Australia	A20-13	2020	N	N	[1]
**10**	*A. caviae*	58	Australia	AF24-2-1	2024	N	N	This study
**11**	*A. caviae*	60	Australia	A20-11	2020	N	N	[1]
**12**	*A. caviae*	61	Australia	A20-3	2020	N	N	[1]
**13**	*A. caviae*	64	Australia	A20-4	2020	N	N	[1]
**14**	*A. caviae*	65	Australia	A21-20	2021	N	N	[1]
**15**	*A. caviae*	66	Australia	A21-17	2021	N	N	[1]
**16**	*A. caviae*	70	Australia	A20-16	2020	N	N	[1]
**17**	*A. caviae*	72	Australia	AF12-9	2024	N	N	This study
**18**	*A. caviae*	73	Australia	AF23-12-8	2023	N	N	This study
**19**	*A. caviae*	76	Australia	A20-18	2020	N	N	[1]

**20**	*A. caviae*	76	Australia	AF23-8-1	2023	N	N	This study
**21**	*A. caviae*	79	Australia	A20-15	2020	N	N	[1]
**22**	*A. caviae*	81	Australia	Aero17	2022	N	N	[1]
**23**	*A. caviae*	82	Australia	A20-9	2020	N	N	[1]
**24**	*A. caviae*	84	Australia	A20-7	2020	N	N	[1]
**25**	*A. caviae*	101	Australia	AF24-5-2	2024	N	N	This study
**26**	*A. dhakensis*	38	Australia	AF24-5-3	2024	P	N	This study
**27**	*A. dhakensis*	41	Australia	A211-6	2021	P	N	This study
**28**	*A. dhakensis*	56	Australia	A21-2	2021	P	N	[2]
**29**	*A. dhakensis*	44	Australia	A25	2019	P	N	[2]
**30**	*A. dhakensis*	54	Australia	AF24-5-1	2024	P	N	This study
**31**	*A. dhakensis*	88	Australia	AF23-6-6	2023	P	N	This study
**32**	*A. hydrophila*	30	Australia	AF22-1	2022	P	P	This study
**33**	*A. hydrophila*	45	Australia	A20-2	2020	N	P	[2]
**34**	*A. hydrophila*	53	Australia	AF24-2-5	2024	N	P	This study
**35**	*A. hydrophila*	52	Australia	A21-3	2021	P	P	[2]
**36**	*A. hydrophila*	57	Australia	A21-7	2021	N	P	[2]
**37**	*A. hydrophila*	58	Australia	A21-21	2021	N	P	[2]
**38**	*A. hydrophila*	61	Australia	A21-9	2021	P	P	[2]
**39**	*A. hydrophila*	61	Australia	AF24-2-4	2024	P	N	This study
**40**	*A. hydrophila*	63	Australia	AF24-2-3	2024	N	P	This study
**41**	*A. hydrophila*	69	Australia	AF23-12-5	2023	P	P	This study
**42**	*A. hydrophila*	72	Australia	AF23-9-1	2023	N	P	This study

**43**	*A. hydrophila*	78	Australia	A21-22	2021	P	P	[2]
**44**	*A. veronii*	14	Australia	A20-12	2020	P	N	[3]
**45**	*A. veronii*	17	Australia	A21-4	2021	P	N	[3]
**46**	*A. veronii*	20	Australia	A21	2021	P	N	[3]
**47**	*A. veronii*	21	Australia	A21-16	2021	P	N	[3]
**48**	*A. veronii*	22	Australia	A21-31	2021	P	N	This study
**49**	*A. veronii*	29	Australia	AF23-6-4	2023	P	N	This study
**50**	*A. veronii*	32	Australia	A211-3	2021	P	N	This study
**51**	*A. veronii*	32	Australia	AF12-1	2024	P	N	This study
**52**	*A. veronii*	34	Australia	AF23-12-2	2023	P	N	This study
**53**	*A. veronii*	37	Australia	A7	2019	P	N	[3]
**54**	*A. veronii*	37	Australia	A29V	2019	P	N	[3]
**55**	*A. veronii*	40	Australia	A21-10	2021	P	N	[3]
**56**	*A. veronii*	40	Australia	AF12-13	2024	P	N	This study
**57**	*A. veronii*	40	Australia	AF24-5-4	2024	P	N	This study
**58**	*A. veronii*	44	Australia	A21-13	2021	P	N	[3]
**59**	*A. veronii*	45	Australia	A21-5	2021	P	N	[3]
**60**	*A. veronii*	46	Australia	A27	2019	P	N	[3]
**61**	*A. veronii*	52	Australia	AF23-10-1	2023	P	N	This study
**62**	*A. veronii*	55	Australia	A21-19	2021	P	N	[3]
**63**	*A. veronii*	56	Australia	A26	2019	P	N	[3]
**64**	*A. veronii*	57	Australia	AF12-6	2024	P	N	This study
**65**	*A. veronii*	58	Australia	A21-11	2021	P	N	[3]

**66**	*A. veronii*	61	Australia	A21-8	2021	P	N	[3]
**67**	*A. veronii*	62	Australia	A20	2020	P	N	[3]
**68**	*A. veronii*	64	Australia	A21-27	2021	P	N	This study
**69**	*A. veronii*	64	Australia	A211-2	2021	P	N	This study
**70**	*A. veronii*	64	Australia	AF23-6-3	2023	P	N	This study
**71**	*A. veronii*	65	Australia	A21-6	2021	P	N	[3]
**72**	*A. veronii*	72	Australia	A21-14	2021	P	N	[3]
**73**	*A. veronii*	77	Australia	AS1	2021	P	N	[4]
**74**	*A. veronii*	79	Australia	AF12-4	2024	P	N	This study
**75**	*A. veronii*	79	Australia	AF23-12-4	2023	P	N	This study
**76**	*A. veronii*	80	Australia	A20-10	2020	P	N	[3]
**77**	*A. veronii*	81	Australia	A20-17	2020	P	N	[3]
**78**	*A. veronii*	81	Australia	AF23-12-7	2023	P	N	This study
**79**	*A. veronii*	82	Australia	A20-14	2020	P	N	[3]
**80**	*A. veronii*	82	Australia	AF23-9-2	2023	P	N	This study
**81**	*A. veronii*	85	Australia	AF23-6-1	2023	P	N	This study
**82**	*A. veronii*	86	Australia	AF23-6-5	2023	P	N	This study
**83**	*A. veronii*	87	Australia	A20-8	2020	P	N	[3]
**84**	*A. veronii*	89	Australia	A9	2019	P	N	[3]
**85**	*A. veronii*	90	Australia	AF23-12-3	2023	P	N	This study
**86**	*A. veronii*	91	Australia	A20-5	2020	P	N	[3]
**87**	*A. veronii*	91	Australia	A21-15	2021	P	N	[3]
**88**	*A. veronii*	92	Australia	A8	2019	P	N	[3]

**89**	*A. allosaccharophila*	51	Australia	A21-32	2021	N	N	This study
**90**	*A. media*	14	Australia	A21-30	2021	N	N	This study
**91**	*A. rivipollensis*	36	Australia	A20-1	2020	P	N	[2]
**92**	*A. rivipollensis*	85	Australia	A20-6	2020	N	N	[2]
**93**	*A. caviae*	1	China	PDAE14025	2014			PubMLST
**94**	*A. caviae*	1	China	PDAE14039	2014			PubMLST
**95**	*A. caviae*	1	China	PDAE14044	2014			PubMLST
**96**	*A. caviae*	1	China	15-21	2015			PubMLST
**97**	*A. caviae*	1	China	15-27	2015			PubMLST
**98**	*A. caviae*	1	China	15-9	2015			PubMLST
**99**	*A. caviae*	2	China	PDAE14043	2014			PubMLST
**100**	*A. caviae*	2	China	18-2	2018			PubMLST
**101**	*A. caviae*	3	China	15-5	2015			PubMLST
**102**	*A. caviae*	4	China	PDAE14040	2014			PubMLST
**103**	*A. caviae*	4	China	16-8	2016			PubMLST
**104**	*A. caviae*	13	China	B2230004	2022			PubMLST
**105**	*A. caviae*	15	China	15-19	2015			PubMLST
**106**	*A. caviae*	24	China	PDAE14045	2014			PubMLST
**107**	*A. caviae*	25	China	15-12	2015			PubMLST
**108**	*A. caviae*	30	China	PDAE14064	2014			PubMLST
**109**	*A. caviae*	30	China	B2330001	2023			PubMLST
**110**	*A. caviae*	33	China	PDAE14006	2014			PubMLST

**111**	*A. caviae*	37	China	B2130008	2021			PubMLST
**112**	*A. caviae*	38	China	B1930011	2019			PubMLST
**113**	*A. caviae*	41	China	B1930003	2019			PubMLST
**114**	*A. caviae*	48	China	B2030005	2020			PubMLST
**115**	*A. caviae*	61	China	15-26	2015			PubMLST
**116**	*A. caviae*	63	China	15-32	2015			PubMLST
**117**	*A. caviae*	65	China	PDAE14052	2014			PubMLST
**118**	*A. caviae*	66	China	15-23	2015			PubMLST
**119**	*A. caviae*	66	China	15-24	2015			PubMLST
**120**	*A. caviae*	66	China	18-6	2018			PubMLST
**121**	*A. caviae*	68	China	15-36	2015			PubMLST
**122**	*A. caviae*	70	China	PDAE14021	2014			PubMLST
**123**	*A. caviae*	75	China	15-28	2015			PubMLST
**124**	*A. caviae*	77	China	PDAE14013	2014			PubMLST
**125**	*A. caviae*	77	China	PDAE14048	2014			PubMLST
**126**	*A. caviae*	77	China	B2130010	2021			PubMLST
**127**	*A. dhakensis*	28	China	16-9	2016			PubMLST
**128**	*A. dhakensis*	64	China	B2030004	2020			PubMLST
**129**	*A. dhakensis*	73	China	16-5	2016			PubMLST
**130**	*A. hydrophila*	1	China	PDAE14003	2014			PubMLST
**131**	*A. hydrophila*	1	China	PDAE14035	2014			PubMLST
**132**	*A. hydrophila*	2	China	PDAE14049	2014			PubMLST
**133**	*A. hydrophila*	2	China	PDAE14049	2014			PubMLST

**134**	*A. hydrophila*	17	China	PDAE14007	2014			PubMLST
**135**	*A. hydrophila*	24	China	PDAE14004	2014			PubMLST
**136**	*A. hydrophila*	27	China	PDAE14060	2014			PubMLST
**137**	*A. hydrophila*	28	China	PDAE14051	2014			PubMLST
**138**	*A. hydrophila*	34	China	B1930012	2019			PubMLST
**139**	*A. hydrophila*	38	China	16-20	2016			PubMLST
**140**	*A. hydrophila*	44	China	PDAE14067	2014			PubMLST
**141**	*A. hydrophila*	45	China	PDAE14047	2014			PubMLST
**142**	*A. hydrophila*	47	China	PDAE14010	2014			PubMLST
**143**	*A. hydrophila*	49	China	PDAE14005	2014			PubMLST
**144**	*A. hydrophila*	51	China	PDAE14066	2014			PubMLST
**145**	*A. hydrophila*	52	China	15-17	2015			PubMLST
**146**	*A. hydrophila*	58	China	PDAE14058	2014			PubMLST
**147**	*A. hydrophila*	60	China	PDAE14033	2014			PubMLST
**148**	*A. hydrophila*	65	China	PDAE14065	2014			PubMLST
**149**	*A. hydrophila*	67	China	PDAE14008	2014			PubMLST
**150**	*A. hydrophila*	83	China	PDAE14031	2014			PubMLST
**151**	*A. veronii*	0.58	China	PDAE14026	2014			PubMLST
**152**	*A. veronii*	1	China	PDAE14034	2014			PubMLST
**153**	*A. veronii*	1	China	15-22	2015			PubMLST
**154**	*A. veronii*	1	China	18-9	2018			PubMLST
**155**	*A. veronii*	2	China	PDAE14015	2014			PubMLST
**156**	*A. veronii*	2	China	PDAE14012	2014			PubMLST

**157**	*A. veronii*	2	China	16-6	2016			PubMLST
**158**	*A. veronii*	2	China	18-3	2018			PubMLST
**159**	*A. veronii*	3	China	15-10	2015			PubMLST
**160**	*A. veronii*	8	China	17-1	2017			PubMLST
**161**	*A. veronii*	11	China	PDAE14041	2014			PubMLST
**162**	*A. veronii*	15	China	B2330002	2023			PubMLST
**163**	*A. veronii*	17	China	B2230013	2022			PubMLST
**164**	*A. veronii*	18	China	15-41	2015			PubMLST
**165**	*A. veronii*	20	China	B2030003	2020			PubMLST
**166**	*A. veronii*	20	China	B2130025	2021			PubMLST
**167**	*A. veronii*	21	China	PDAE14069	2014			PubMLST
**168**	*A. veronii*	21	China	15-20	2015			PubMLST
**169**	*A. veronii*	21	China	B1930010	2019			PubMLST
**170**	*A. veronii*	21	China	B2130003	2021			PubMLST
**171**	*A. veronii*	22	China	PDAE14019	2014			PubMLST
**172**	*A. veronii*	22	China	16-12	2016			PubMLST
**173**	*A. veronii*	23	China	PDAE14068	2014			PubMLST
**174**	*A. veronii*	25	China	PDAE14002	2014			PubMLST
**175**	*A. veronii*	25	China	PDAE14061	2014			PubMLST
**176**	*A. veronii*	25	China	16-17	2016			PubMLST
**177**	*A. veronii*	26	China	B2130022	2021			PubMLST
**178**	*A. veronii*	27	China	PDAE14020	2014			PubMLST
**179**	*A. veronii*	27	China	18-1	2018			PubMLST

**180**	*A. veronii*	27	China	B2130013	2021			PubMLST
**181**	*A. veronii*	27	China	B2230008	2022			PubMLST
**182**	*A. veronii*	28	China	15-34	2015			PubMLST
**183**	*A. veronii*	28	China	17-6	2017			PubMLST
**184**	*A. veronii*	28	China	B2230003	2022			PubMLST
**185**	*A. veronii*	28	China	B2230005	2022			PubMLST
**186**	*A. veronii*	29	China	PDAE14024	2014			PubMLST
**187**	*A. veronii*	29	China	PDAE14059	2014			PubMLST
**188**	*A. veronii*	29	China	16-11	2016			PubMLST
**189**	*A. veronii*	29	China	17-5	2017			PubMLST
**190**	*A. veronii*	30	China	15-35	2015			PubMLST
**191**	*A. veronii*	30	China	15-37	2015			PubMLST
**192**	*A. veronii*	30	China	18-10	2018			PubMLST
**193**	*A. veronii*	31	China	PDAE14001	2014			PubMLST
**194**	*A. veronii*	31	China	PDAE14037	2014			PubMLST
**195**	*A. veronii*	32	China	PDAE14018	2014			PubMLST
**196**	*A. veronii*	32	China	PDAE14029	2014			PubMLST
**197**	*A. veronii*	32	China	15-33	2015			PubMLST
**198**	*A. veronii*	32	China	16-7	2016			PubMLST
**199**	*A. veronii*	32	China	17-4	2017			PubMLST
**200**	*A. veronii*	33	China	PDAE14014	2014			PubMLST
**201**	*A. veronii*	33	China	PDAE14042	2014			PubMLST
**202**	*A. veronii*	33	China	PDAE14050	2014			PubMLST

**203**	*A. veronii*	33	China	PDAE14053	2014			PubMLST
**204**	*A. veronii*	33	China	15-42	2015			PubMLST
**205**	*A. veronii*	34	China	15-25	2015			PubMLST
**206**	*A. veronii*	35	China	PDAE14054	2014			PubMLST
**207**	*A. veronii*	35	China	B2230010	2022			PubMLST
**208**	*A. veronii*	35	China	B2330003	2023			PubMLST
**209**	*A. veronii*	36	China	15-11	2015			PubMLST
**210**	*A. veronii*	37	China	B2130023	2021			PubMLST
**211**	*A. veronii*	39	China	15-29	2015			PubMLST
**212**	*A. veronii*	42	China	PDAE14016	2014			PubMLST
**213**	*A. veronii*	42	China	B2130014	2021			PubMLST
**214**	*A. veronii*	43	China	PDAE14046	2014			PubMLST
**215**	*A. veronii*	43	China	B2130020	2021			PubMLST
**216**	*A. veronii*	45	China	B1930005	2019			PubMLST
**217**	*A. veronii*	47	China	PDAE14057	2014			PubMLST
**218**	*A. veronii*	48	China	15-8	2015			PubMLST
**219**	*A. veronii*	50	China	PDAE14009	2014			PubMLST
**220**	*A. veronii*	51	China	PDAE14017	2014			PubMLST
**221**	*A. veronii*	51	China	PDAE14038	2014			PubMLST
**222**	*A. veronii*	52	China	PDAE14011	2014			PubMLST
**223**	*A. veronii*	52	China	16-13	2016			PubMLST
**224**	*A. veronii*	52	China	16-2	2016			PubMLST
**225**	*A. veronii*	53	China	PDAE14027	2014			PubMLST

**226**	*A. veronii*	54	China	15-1	2015			PubMLST
**227**	*A. veronii*	56	China	16-3	2016			PubMLST
**228**	*A. veronii*	56	China	17-7	2017			PubMLST
**229**	*A. veronii*	57	China	PDAE14032	2014			PubMLST
**230**	*A. veronii*	58	China	PDAE14062	2014			PubMLST
**231**	*A. veronii*	58	China	15-30	2015			PubMLST
**232**	*A. veronii*	58	China	15-40	2015			PubMLST
**233**	*A. veronii*	59	China	PDAE14056	2014			PubMLST
**234**	*A. veronii*	59	China	PDAE14073	2014			PubMLST
**235**	*A. veronii*	59	China	16-16	2016			PubMLST
**236**	*A. veronii*	60	China	PDAE14028	2014			PubMLST
**237**	*A. veronii*	60	China	PDAE14071	2014			PubMLST
**238**	*A. veronii*	60	China	18-5	2018			PubMLST
**239**	*A. veronii*	62	China	18-8	2018			PubMLST
**240**	*A. veronii*	62	China	B2030002	2020			PubMLST
**241**	*A. veronii*	63	China	15-38	2015			PubMLST
**242**	*A. veronii*	63	China	15-39	2015			PubMLST
**243**	*A. veronii*	63	China	15-7	2015			PubMLST
**244**	*A. veronii*	63	China	16-14	2016			PubMLST
**245**	*A. veronii*	63	China	17-2	2017			PubMLST
**246**	*A. veronii*	64	China	PDAE14055	2014			PubMLST
**247**	*A. veronii*	64	China	15-13	2015			PubMLST
**248**	*A. veronii*	64	China	18-4	2018			PubMLST

**249**	*A. veronii*	65	China	PDAE14022	2014			PubMLST
**250**	*A. veronii*	65	China	PDAE14036	2014			PubMLST
**251**	*A. veronii*	65	China	16-22	2016			PubMLST
**252**	*A. veronii*	67	China	PDAE14072	2014			PubMLST
**253**	*A. veronii*	70	China	PDAE14070	2014			PubMLST
**254**	*A. veronii*	72	China	PDAE14030	2014			PubMLST
**255**	*A. veronii*	72	China	17-3	2017			PubMLST
**256**	*A. veronii*	76	China	PDAE14023	2014			PubMLST
**257**	*A. veronii*	80	China	PDAE14063	2014			PubMLST
**258**	*A. veronii*	85	China	16-19	2016			PubMLST
**259**	*A. media*	30	China	B2230001	2022			PubMLST
**260**	*A. rivipollensis*	16	China	B2230007	2022			PubMLST
**261**	*A. rivipollensis*	60	China	B2230006	2022			PubMLST
**262**	*A. trota*	29	China	15-31	2015			PubMLST
**263**	*A. trota*	30	China	16-23	2016			PubMLST
**264**	*A. trota*	31	China	15-14	2015			PubMLST
**265**	*A. trota*	32	China	15-18	2015			PubMLST
**266**	*A. trota*	45	China	B1930006	2019			PubMLST

The presence of *aerA* and *ast* genes were examined in 92 *Aeromonas* strains isolated from patients with gastroenteritis in Australia. P: positive. N: negative.

References

1. Chong, S. K. T. et al. Analysis of global *Aeromonas caviae* genomes revealed that strains carrying T6SS are more common in human gastroenteritis than in environmental sources and are often phylogenetically related. *Microbial Genomics* **10**, 129 (2024).
2. Yuwono, C. et al. The Isolation of *Aeromonas* Species and Other Common Enteric Bacterial Pathogens from Patients with Gastroenteritis in an Australian Population. *Microorganisms* **9**, 1440 (2021).
3. Liu, F. et al. Analysis of global *Aeromonas veronii* genomes provides novel information on source of infection and virulence in human gastrointestinal diseases. *BMC Genomics* **23**, 1-15 (2022).
4. Lee, S.A. et al. Emerging *Aeromonas* enteric infections: their association with inflammatory bowel disease and novel pathogenic mechanisms. *Microbiology Spectrum* **11**, e0108823 (2023)
